# Efforts and Challenges to Ensure Continuity of Mental Healthcare Service Delivery in a Low Resource Settings During COVID-19 Pandemic—A Case of a Kenyan Referral Hospital

**DOI:** 10.3389/fpsyt.2020.588216

**Published:** 2021-01-14

**Authors:** Edith Kwobah, Florence Jaguga, Kiptoo Robert, Elias Ndolo, Jane Kariuki

**Affiliations:** ^1^Department of Mental Health, Moi Teaching and Referral Hospital, Eldoret, Kenya; ^2^Mental Health and Rehabilitative Services, Moi Teaching and Referral Hospital, Eldoret, Kenya

**Keywords:** mental health care, low resource setting, Kenya, Moi Teaching and Referral Hospital, continuity of care

## Abstract

The rising number of patients with Covid-19 as well as the infection control measures have affected healthcare service delivery, including mental healthcare. Mental healthcare delivery in low and middle income countries where resources were already limited are likely to be affected more during this pandemic. This paper describes the efforts of ensuring mental healthcare delivery is continued in a referral hospital in Kenya, Moi Teaching and Referral hospital, as well as the challenges faced. These efforts are guided by the interim guidelines developed by the Kenyan ministry of health. Some of the adjustments described includes reducing number of patients admitted, shortening the stay in the inpatient setting, using outdoors for therapy to promote physical distancing, utilization of electronic platforms for family therapy sessions, strengthening outpatient services, and supporting primary care workers to deliver mental health care services. Some of the challenges include limited ability to move about, declining ability for patients to pay out of pocket due to the economic challenges brought about by measures to control Covid-19, limited drug supplies in primary care facilities, inability to fully implement telehealth due to connectivity issues and stigma for mental health which results in poor social support for the mentally ill patients. It is clear that current pandemic has jeopardized the continuity of usual mental healthcare in many settings. This has brought to sharp focus the need to decentralize mental health care and promote community based services. Meanwhile, there is need to explore feasible alternatives to ensure continuity of care.

## Introduction

The Severe Acute Respiratory Syndrome Coronavirus (SARS-CoV-2) also known as COVID-19 pandemic has infected millions of people globally and claimed thousands of lives. It also has affected various aspects of societies way of doing business, disrupting the lives of everyone, regardless of age, gender, or race (1). Because of the highly contagious nature of COVID-19 through close contacts (2), several measures have been put in place to limit the spread of the virus (3). These measures as is now widely known include staying at home, wearing masks, limited public transport, and closing of business (4). These measures are key in not only reducing the spread but also reducing the percentage of people who will require hospitalization as this would otherwise overwhelm the healthcare systems (5).

The growing numbers of COVID-19 patients as well as the measures that have been put in place have had a significant bearing on access to general health care (6), as well as mental healthcare (7). For example, the directives to stay home, and keep physical distance has disrupted previous ease of traveling from one place to another, including traveling to a health facility. In addition, there is stigma and fear related to the Covid-19 infections and this includes fear of hospitals which are seen as high risk areas (8). Further, with the growing numbers of COVID-19 infected patients, a lot of material and human resources have been directed toward fighting the pandemic, potentially straining delivery of services for other medical conditions. The effect of the measures that demand reduced movement is likely more severe for patients with chronic diseases like mental illness who require regular follow up, check up, and prescription refills (9). This is especially tough for systems in Low and Middle income countries (LMIC) which were already suffering from inadequate resources and capacity even before Covid-19 due to limited resources for mental health (10).

Kenya has not been spared in this struggle and since the first patient tested positive in March 2020, the number of infected citizens has been on the rise with more than 18,000 positive patients and the death toll steadily rising, standing at 285 deaths at the end of July. This has resulted in wide spread anxiety on how the hospitals would address the risk posed by huge numbers of people coming into the health facilities. Earlier in the pandemic, one of the recommendations was to minimize the number of patients coming to hospitals in order to decongest the facilities and reduce risk of infection, but this changed when there were reports of patients with chronic illness complicating at home due to inability to access care (11). In addition, the pandemic has likely altered the health seeking behavior due to several pandemic related factors. For example, a study done in slum communities of Bangladesh, Kenya, Nigeria and Pakistan points to a reduction in general health seeking behavior due to factors such as increased cost of healthcare, reduced household income, increased challenges in physically reaching healthcare facilities, and exacerbated reluctance of residents to seek healthcare due to fear of infection and stigmatization (12).

This pandemic, therefore, calls professionals, including mental health practitioners to identify and problem-solve challenges that have a bearing on the service delivery, in order to minimize negative outcomes (13). It is clear that provision of mental healthcare has to be adjusted to meet the need of patients (14). There have been several suggestions of what need to be done to meet this need, but such suggestions such as use of telehealth that that are being implemented in developed countries (15) are not easy in low resource settings such as Kenya. This paper highlights adjustments that has been made in order to ensure continuity of mental healthcare in low resource settings, citing experiences from one of the leading mental health facilities in Kenya, the Moi Teaching and Referral Hospital (MTRH). It also points out some of the challenges faced and gives recommendations for possible way forward.

## Efforts to Ensure Continuity of Mental Healthcare in Low Resource Settings

### The Kenyan Context

Kenya is a LMIC country with a population of about 48 million people according to the 2019 census (16). A study in a community sample in Western Kenya showed that 45% of the community have at least one mental health disorder (17), which was very close to an earlier study among patients attending general hospitals which reported a prevalence of 42% (18). There is also a huge shortage of human resources for mental health as only 29 of the 3,956 government owned facilities in Kenya actually provide mental health care, with a gross shortage of mental health workers in the country, hence patient travel long distances to access care (19). The cost of care is further complicated by the fact that <20% of the population has insurance cover (20), hence most patients pay out of pocket for their mental health services (21).

In March, the Kenyan government announced several precautionary measures which included a dusk to dawn curfew, wearing of masks in public places, closure of high risk businesses, and reduction of passengers in the public transport vehicles. The ministry of health Kenya recognized that mentally ill persons formed a population of vulnerable groups and their care during this pandemic would require special consideration. A protocol to offer guidelines on continued management of stable patients, admission of patients, management of mentally ill persons who tested positive for COVID-19, and the mental well-being of healthcare workers was developed, led by the Kenyan division of Mental Health (22). The document contains provisions that give guidance on the delivery of mental health services for persons with newly diagnosed and pre-existing mental disorders during the pandemic, including guidelines on the utilization of tele-health methods as well as the delivery of mental health care for persons in isolation and quarantine.

### Efforts at a National Referral Hospital in Kenya to Ensure Continuity of Care

Moi Teaching and Referral Hospital is the second largest national referral center in Kenya located in Uasin Gishu County (http://www.mtrh.go.ke/). The Hospital serves residents of Western Kenya Region (representing at least 22 Counties), parts of Eastern Uganda and Southern Sudan with a population of ~24 Million. MTRH has one of the best mental health facilities in Kenya, having a purposely built 70 bed capacity in-patient ward (Mental Health Unit-MHU) and 16 bed capacity Alcohol and Drug Abuse unit (ADAR). In addition, MTRH has robust outpatient services comprised of a daily walk in service located at the accident and emergency department that is open every day of the week and is the main entry into the mental health service, a weekly (Monday) clinic that serves children, forensic clients, and the addiction clients and a weekly (Wednesday) clinic that serves all the other general psychiatry patients.

Despite the challenges that Covid-19 brought to the delivery of health services, the MTRH mental health department needed to ensure the continuity of mental health services remain open for a number of reasons. First, the department serves patients from a huge catchment area in Western Kenya that do not have a psychiatrist or even a mental health service (there only 8 psychiatrists and <30 psychiatry nurses) available in the entire region. Changing our service delivery means an alternative service in peripheral hospitals which would not be feasible in the very short term. Second, due to availability of subsidized drugs that have been made possible by MTRH partners (Olanzapine and Fluoxetine are donated by El Lilly pharma making them affordable for many poor Kenyans, hence widely prescribed) patients prefer to travel long distances to lower the cost of purchasing medications. Changing our services would mean several patients going without medication as these two drugs are not only expensive but also not stocked in most peripheral hospitals. Third due to stigma related to mental illness other service providers in primary care setting may not be comfortable managing mentally ill patients and this may mean that in the context of widespread COVID-19 related anxiety among health workers there may be less accommodation for these patients. Fourth, persons with mental disorders often face difficulties accessing general healthcare and in the context of Covid-19, cognitive impairments may affect their ability to adhere to precautionary measures hence increase their risk of contracting COVID. Lack of care would ultimately increase the patient risk of contracting COVID-19 as well as other risk associated with poorly controlled mental illness.

We made specific adjustments to ensure that as we served patients, we also protected both staff and patients from contracting COVID-19:

1. At the alcohol and drug abuse unit we reduced the capacity of the clients from 16 to a maximum of 8 to allow for physical distancing. At the same time, we encouraged more patients to get booked in the outpatient alcohol and drug abuse clinic to cater for those who were locked out of the inpatient care. We held sessions with patients to sensitize them on infection control measures. Within the inpatient unit, we put the patients into smaller groups for group therapy sessions. We required that most of the occupational therapy activities are conducted outdoors which allows for more physical space and also allows better circulation given that the current unit is quite small. We also reverted to family therapy sessions using online platforms to minimize in person meetings and need to travel long distances.
2. In the mental health unit we limited admissions to only those who are severely agitated. We strengthened triage in our daily outpatient walk in service to ensure that all those who could be managed at home were not admitted but were instead given a shorter review date. This was done by ensuring that a senior resident doctor reviewed all the patients intended for admissions. We shortened the stay in mental health inpatient care by ensuring frequent reviews and allowing patients home as soon as there was some improvement, as opposed to full recovery. We educated the families through brief family therapy session, to accept supporting their kin at home even when the mental health symptoms were not fully resolved, as long as there was no threat to self or others.
3. To increase staff preparedness and also reduce anxiety, we trained the mental healthcare providers on infection controls measures, and how to screen for COVID-19, using the Ministry of health Case definition. We also set aside space for any mentally ill patients who displayed symptoms of COVID-19. Further, we put efforts to continually provide mental health and psychosocial support to our staff to continually allay their anxiety—through both group and individual therapy sessions.
4. We continued to empower primary care workers in peripheral hospitals to manage mentally ill patients, in-order to cater for those who would not get to MTRH. This we did by conducting a 1 week zoom training of the Mental Health Gap Intervention Program MHGAP (23), but also supporting primary care workers through on phone consultations with a psychiatrist. We also supported continuation of alcohol support groups in the community through phone calls. This would ensure continuity of care for those who could not be accommodated in the inpatient service. Capacity building and community based alcohol support groups was made possible by an ongoing collaboration between MTRH and Indiana University which is currently funded by the Astellas Global Health Foundation.
5. We also allocated a mental health nurse to make a telephone call to clients who did not come for their follow-up clinic using the phone number included in the hospital records, to explain to them the need to adhere to treatment as well as help them problem solve to ensure they continued with medication in whatever circumstances they were in that hindered in—person attendance. The nurse made the call in a private consultation room to safeguard privacy of the patient.
6. We also continued to promote mental wellness among staff and the general public by incorporating mental wellness into all Covid-19 related trainings within MTRH, as well as participating in media sensitizations on local media stations, as well as participating in various webinars targeting various healthcare providers.

**Figure 1:**
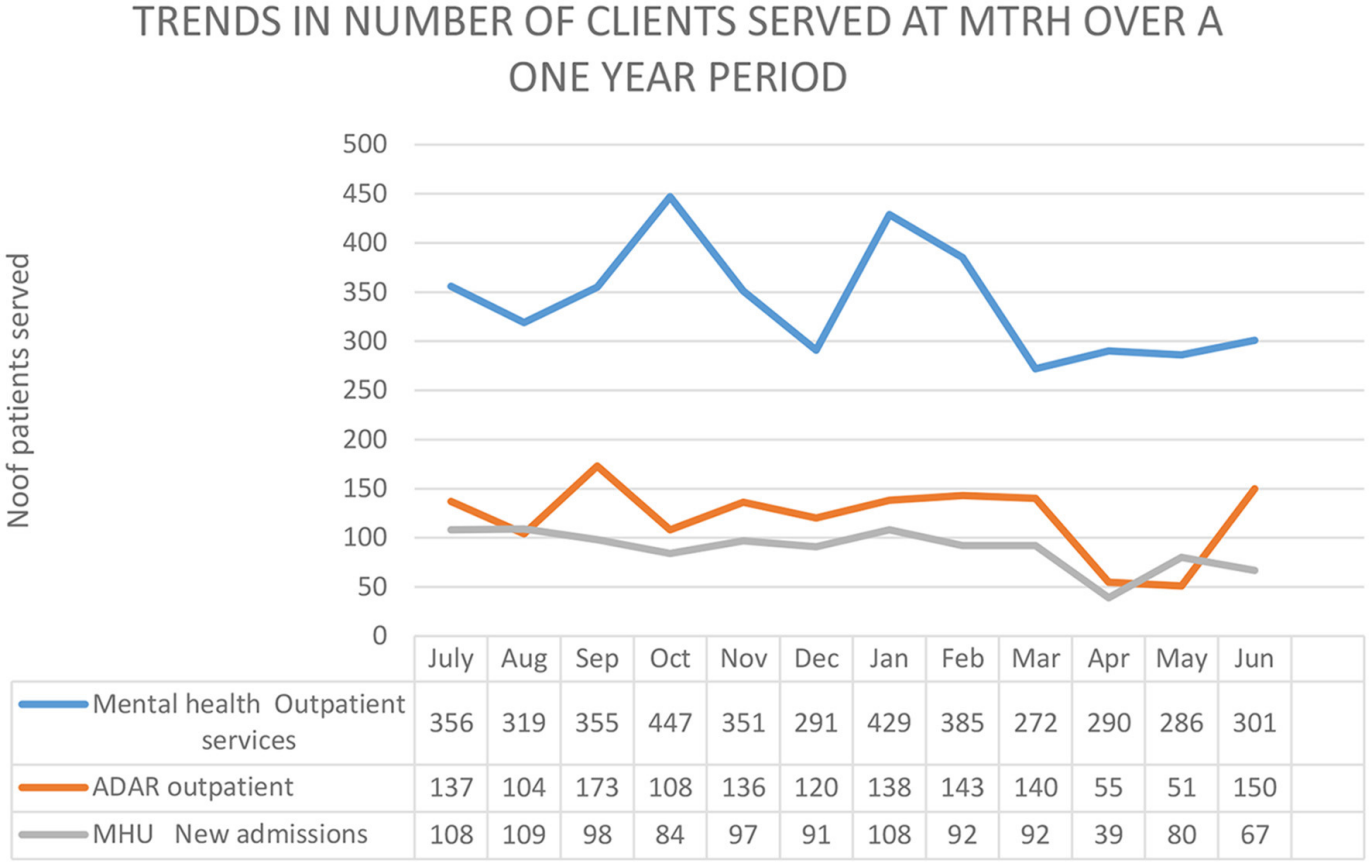
Showing utilization of selected mental health service between July 2019 and June 2020. ADAR, Alcohol and drug abuse—Rehabilitation; MHU, Mental health Unit.

Figure 1 shows the trends of selected mental health service during the Covid-19 season at MTRH. The figure shows some reduction in admissions into mental health unit in April, the slight decline in number of patients attending mental health clinic in March, April, and May and the decline in attendance to alcohol and drug abuse outpatient clinic in April, May. The numbers then begin to be restored, and we attribute this in part to our efforts encouraging patients to adhere to care.

### Challenges Faced in Ensuring Continuity of Care

In the context of COVID-19 pandemic, several factors contribute to disruption in ensuring continuity of mental healthcare in a low resource setting such as ours. These include:

*Limited ability to move about:* Patients served at MTRH travel long distances, some farther than 300 km on road. Using public transport, this would require use of two or three public vehicles, which means a lot of time spent during the connections and waiting for the vehicle to fill with passengers. In Kenya, public vehicles leave the bus stage upon filling up to capacity rather than following a time schedule. When COVID-19 measures were announced by the government, patients who needed to travel long distances to hospital were affected. When contacted, some patients reported being afraid of the journey as they would be caught on the road when the dusk to dawn curfew set in. In addition the cost of traveling had gone up as public vehicles almost doubled the bus fares as they could only carry three quarters of their usual capacity in order to allow physical distancing. Further due to the high cost of traveling, the number of people traveling had reduced, which means even more time waiting for the public vehicles to get filled.

*Declining social economic status:* Similar to other low resource settings the economic breakdown due to job losses has a negative effect on families ability to support costs related to mental health care (24). Because many of our patients have to pay out of pocket for their mental health care, this is likely to be affected greatly in a situation where spending on medication determines whether people will have food on the table (25). Given that mild to moderate mental illness is not seen as life threatening, it is likely that a family struggling to feed their children would not see care for mental illness as a priority.

*Stigma associated with mental illness leading to poor social support*. Discrimination and rejection of mentally ill patients is a major obstacle to care especially in low resource settings such as Kenya. During this season, we have had patients abandoned in the mental health unit by their loved ones due to the aggressive behaviors manifested in acute phases. This has led to patients staying longer than desired, hence making it difficult to achieve the desired numbers for physical distance.

*Limited drug supply in peripheral facilities:* Unlike other medications, the supply of even basic mental health drugs in most peripheral hospitals is largely limited in Kenya. This has resulted from the inadequate integration of mental healthcare in primary care setting. This has resulted in a limited prescription of mental health drugs and because most hospitals in Kenya use the pull system where you stock what is largely prescribed, the drugs are largely not procured. This means that a patient visiting any primary care facility without a mental health worker is immediately referred, and in this case MTRH is the main referral hospital.

*Low internet availability for tele-health:* Due to the effect of COVID-19 on ambulation, use of electronic platforms to deliver mental healthcare has been suggested as an effective alternative (26). In this circumstance, one would have expected that telehealth would somewhat bridge the gap for care in the setting of MTRH, as has been documented in developed countries such a Australia (27). Although many Kenyans own a phone, there is the cost of airtime and bundles to support teleconsultation. This is not an easy option for many poor clients, and it is further complicated by poor network connectivity in many remote areas.

*Difficulties of mentally ill patients to adhere with infection control measures:* There is evidence indicating that psychiatric inpatients may be more susceptible to COVID_19 compared to other patients in other health facilities because they often share common therapy rooms, dining, and bathroom spaces, which increase patient to patient contact (28). In addition impaired mental state, poor self-control and self-care, and lack of insight, mentally ill patients have challenges practicing infection control measures to protect themselves such as hand hygiene and wearing of masks. According to the hospital records, most admissions comprise of severely ill patients with Schizophrenia and bipolar mood disorders, who often do not understand the need to wear masks or keep a distance. This has resulted in persistent fear of infection among staff and patients which is worsened by the limited testing capacity meaning that we do not have the luxury of testing each patient who comes into the unit.

*Staff Shortages:* There is a general shortage of mental health care providers in many low and middle income countries. This is the situation even in Kenya and MTRH. We have had instances of quarantining of staff exposed to confirmed patients with COVID-19. When this has occurred, staffing shortages with increased workload and fatigue for the remaining staff has been experienced.

*Limited access to addiction services:* In order to ensure adequate distancing, the unit only admits eight persons at a time. This has resulted in long waiting times for patients requiring specialized treatment for substance use disorder.

### Suggested Way Forward

Governments should put all efforts to ensure that mental healthcare is minimally disrupted during this season given the ramifications of untreated mental illness. First, they could consider providing a financial support, though a mental health stimulus package in order to enable the institutions and families meet the rising cost of care. This we believe is possible as it has been done in other sectors that have been deemed in need by the government. For example, the government of Kenya was able to promote maternal and child health by investing in a free maternal health program in the country which resulted in more mothers accessing care (29) demonstrating that cost is a major hindrance to seeking care. Second they should explore ways of providing basic mental health care in primary care facilities which are more accessible to communities, in order to reduce the need to travel over long distance (30). The need for community based mental healthcare has been suggested as one of the ways to increase access to care especially in rural in Africa as this reduces the need to travel over long distances (31). For this to take place in a context with gross shortage of human resource, there will be need to consider supporting primary care workers to provide care while providing them with adequate training and continuous supervision (32). Facilitating short trainings to enable healthcare workers in peripheral hospitals deliver basic mental health care can reduce the need to refer everyone to major hospitals which are already burdened by COVID-19. Third they should explore ways of strengthening community pharmacists who may play a key role not only in dispensing medication in spaces within the community but also monitoring patient symptoms, hence reducing the burden in major hospitals (33). Fourth, there is need to explore ways of partnering with other stakeholders to increase access to mental health care. These includes partnerships between the public, government owned healthcare service working with Non-Governmental organizations as well as private facilities to promote care. Such a comprehensive partnership model was demonstrated in Nepal when *Possible* an NGO partnered with various public and community based institutions as well as academic institutions, to deliver quality, culturally acceptable mental health care to many otherwise unreached people in rural Nepal (34).

## Conclusion

COVID-19 pandemic has disrupted continuity of mental healthcare especially in low resource settings, and this is likely to affect outcomes. It has brought to sharp focus the need to decentralize mental health care and promote community based services. In the mean time Institutions and governments have a duty to put efforts to minimize interruption of care while exploring feasible alternatives to major hospitals whose access is complicated by the current pandemic.

## Data Availability Statement

The original contributions presented in the study are included in the article/supplementary material, further inquiries can be directed to the corresponding author/s.

## Author Contributions

EK, FJ, and JK conceptualized the manuscript. EK drafted the manuscript. EN collected the data on the trends of service utilization. KR provided information on reasons patients gave for not coming to clinic, as he was the one contacting them. All authors revised the manuscript and approved submission.

## Conflict of Interest

The authors declare that the research was conducted in the absence of any commercial or financial relationships that could be construed as a potential conflict of interest.
